# Confirmation through Genetic Analysis of the Existence of Many Local Phyloclades of the Genus *Simocephalus* (Crustacea, Cladocera) in China

**DOI:** 10.1371/journal.pone.0112808

**Published:** 2014-11-13

**Authors:** Xiaona Huang, Xinlu Shi, Alexey A. Kotov, Fukang Gu

**Affiliations:** 1 School of Life Science, East China Normal University, Shanghai, China; 2 Hangzhou Key Laboratory for Animal Adaptation and Evolution, Hangzhou Normal University, Hangzhou, China; 3 A.N. Severtsov Institute of Ecology and Evolution, Leninsky Prospect 33, Moscow, Russia; Australian Museum, Australia

## Abstract

Previously, a series of *Simocephalus* taxa (Cladocera: Daphniidae) from China were described. Most were proposed to be junior synonyms in the last revision of the genus. Using original material from China and data from GenBank, we investigate the biodiversity and phylogeny of *Simocephalus* using sequences of the cytochrome *c* oxidase subunit I (COI) and the nuclear 18S genes. In both cases, neighbor-joining, maximum likelihood and Bayesian inference analyses led to highly congruent tree topologies. The grouping of the deeper clades agrees with the inter-generic classification of Orlova-Bienkowskaja (2001). Only the populations of *S. serrulatus* from Eurasia and North America seem to be closely related, and there are no other shared species between the two continents. Our study unambiguously confirms the existence of many lineages from the subgenera of *Simocephalus* (*Echinocaudus*) and *Simocephalus* s.str. in China, but their morphology needs to be reexamined by taking a wider range of characters (e.g., of female thoracic limbs and adult males) into consideration.

## Introduction

Cladocera (Crustacea: Branchiopoda) is an important group of micro-crustaceans predominantly inhabiting continental water bodies of different, if not all, types [1]. Among the most famous peculiarities of these animals are their sexually produced diapausing eggs, which are resistant to desiccation and other unfavourable conditions and are important propagules for passive dispersal by different modes, i.e. by birds [1], [2]. Their strong ability to survive passive dispersal was one reason why cladoceran species’ distributions were for a long time accepted as cosmopolitan, but since the 1970’s this concept has changed radically to the so-called non-cosmopolitanism, or “continental endemism” [3], [4], [5], [6]. The correctness of this idea is now confirmed for some genera and species groups [6], [7], [8], [9], although the real diversity and distribution of taxa in other groups needs to be accurately studied.

Some cladocerans, such as species of the genus *Simocephalus* Schödler, 1858 (family Daphniidae Straus, 1820), are used as environmental indicators and “standard” test objects in toxicological studies [10], [11]. Representatives of this genus are very common in vegetation, the open littoral zones of ponds and lakes, the semi-static affluents of rivers and pools and puddles of various types. Based on morphological characters, Orlova-Bienkowskaja [9] recognized 20 valid species in this genus belonging to five subgenera: *Simocephalus* s. str., *Simocephalus* (*Coroncephalus*), *Simocephalus* (*Acutirostratus*), *Simocephalus* (*Echinocaudus*), and *Simocephalus* (*Aquipiculus*). Many of the taxa were regarded by Orlova-Bienkowskaja [9] as junior synonyms of species described earlier.

Several species of *Simocephalus* were identified and then re-described by Chinese authors [12], [13], [14], [15], [16], [17], [18]. Only *Simocephalus heilongjiangensis* Shi & Shi, 1994, which is widely distributed in the tropics, was regarded as a valid species by Orlova-Bienkowskaja [9]. Among the taxa synonymized by Orlova-Bienkowskaja [9], there were several, earlier-described species from China, such as *S. himalayensis* Chiang & Chen, 1974 and *S. beianensis* Shi & Shi, 1994. In addition, *S. himalayensis microdus* Chen, Shi & Shi, 1992 was not discussed by Orlova-Bienkowskaja [9], and its taxonomic status remains unclear. Therefore, there is a conflict that needs to be resolved in the understanding of the taxonomy of the genus between Western investigators, who mainly follow Orlova-Bienkowskaja [9], and Chinese researchers.

Near the end of the 20^th^ century, a powerful new tool for testing taxonomic hypotheses, molecular phylogenetics, became available. In cladocerans, it was mainly applied to species of different *Daphnia* groups [5], [19]. However, molecular phylogenetic studies were subsequently conducted for some other genera and families of the cladocerans [6], [20], [21], [22].

COI barcoding studies for the *Simocephalus* genus were started by Elías-Gutiérrez et al. [23]. These authors recognized eight taxa in tropical Mexico and Guatemala, including two species that are habitually similar to *S. mixtus*, two species habitually similar to *S. exspinosus*, and two species similar to *S. punctatus*. Then, Jeffrey et al. [24] detected six species in Arctic Canada including two different clades of “*S*. cf. *serrulatus*” and four clades of “*S*. cf. *punctatus*”. Young et al. [25], in contrast, found that all of the populations from Taiwan classified as *S. vetulus*, *S. vetuloides* and *S. mixtus* actually belonged to a single species, which compromises the taxonomy according to Orlova-Bienkowskaja [9].

The aim of this paper was to investigate the biodiversity and phylogeny of *Simocephalus* in China using the sequences of cytochrome *c* oxidase subunit I (COI) and nuclear 18S genes.

## Materials and Methods

### Sampling and diagnosis

Specimens were preserved in absolute ethanol (100%) or were brought to the laboratory alive. They were initially examined using a Leica DM 6000 B Digital-Microscope (Germany) with a CTR6000 electric cabinet, Leica LAS software, and Leica DFC 495 CCD. The determination was first made by following Orlova-Bienkowskaja [9]. However, populations were then differentiated according to their morphological characters as proposed in the Chinese literature [13], [16], [17], [18]. The specimens from the studied populations were deposited in the collection of the Hydrobiology Laboratory in Hangzhou Normal University (HZNU), Zhejiang province, People's Republic of China (Table 1).

**Table 1:** The catalogue numbers and the collection location of the specimens from the Hydrobiology Laboratory at Hangzhou Normal University (HZNU), Zhejiang province, People's Republic of China.

Species	Locality	Latitude, longitude,and altitude	GenBank accessionnumbers for COI	GenBank accessionnumbers for 18S	Collectioncatalog numbers
*Simocephalus* cf. *vetulus*	Tongzigou, Muleng, Heilongjiangprovince, China	N44°23.119′,E130°27.464′, 516 m	KF960106, KF960107,KF960109, KF960110	–	YN2011080302
*Simocephalus* cf. *vetulus*	Shangshan village, Fuyang, Zhejiangprovince, China	N30°07.599′,E119°46.746′, 98 m	KF960108	KJ775008	YN2009040508
*Simocephalus* cf. *vetulus*	Boyang lake in Jiangxiprovince, China	N28°57.402′,E117°05. 608′	KF960103-KF960105	–	YN2010103002
*Simocephalus beianensis*	Near the railway station of Beian,Heilongjiang province, China	N48°13. 730′,E126°29. 123′	KF960093-KF960097	KJ775010	YN2013082905
*Simocephalus vetuloides*	Shangshan village, Fuyang,Zhejiang province, China	N30°07. 599′,E119°46. 746′, 98 m	KF960098	KJ775013	YN2009040505
*Simocephalus vetuloides*	Tongzigou, Muleng, Heilongjiangprovince, China	N44°23.119′,E130°27. 464′, 516 m	KF960099	–	YN2011080301
*Simocephalus himalayensis*	The wetland in plateau fromXizang, China	N30°42. 442′,E090°53.284′, 4746 m	KF960070-KF960078	KJ775015	YN20130617
*Simocephalus* cf. *congener*	Sognsvan Lake, Norway	N59°97.088′,E10°73. 109′	KF960053-KF960058	KJ775017	YN2012080501
*Simocephalus himalayensis microdus*	Longhe farm in Heilongjiangprovince, China	N53°20. 322′,E123°58.347′, 376 m	KF960059-KF960063	KJ775022	YN2013082802
*Simocephalus himalayensis microdus*	Harbin Normal University,Heilongjiang province, China	N45°33.597′,E126°41.249′, 98 m	KF960064-KF960069	–	YN2009100401
*S. sp. = Simocephalus serrulatus*in Young et al. 2012	Shangshan village, Fuyang,Zhejiang province, China	N 30°00.038′,E119°45.731′, 43 m	–	KJ775022	YN2009040510
*Simocephalus sibiricus*	Boyang lake, Jiangxiprovince, China.	N28°57.402′,E117°05.608′	KF960086-KF960088	–	YN2010103004
*Simocephalus sibiricus*	Qilin mountain, Jiangxiprovince, China	N29°05.280′,E116°45.333′	KF960079-KF960082, KF960089	–	YN201010300805
*Simocephalus sibiricus*	Haining of Zhejiang province, China	N30°25.816′,E120°26.776′	KF960083-KF960085	KJ775025	YN2012051201
*Simocephalus heilongjiangensis*	Linhai reservoir in Heilongjiang province, China	N45°34.008′,E126°48.081′, 8 m	KF960090- KF960092	KJ775026	YN2011080701
*Simocephalus serrulatus*	Longhe farm in Heilongjiangprovince, China	N53°16.961′,E123°39.251′, 267 m	KF960100- KF960101	–	YN2013082801
*Simocephalus serrulatus*	Zhoushan in Zhejiangprovince, China	N29°56.666′,E122°24.406′, 2 m	KF960102	KJ775028	YN20100606
*Daphnia* cf. *similoides*	Longhe farm in Heilongjiangprovince, China	N53°16′.961′,E123°39.251′, 267 m	KF960111	–	YN2013082802
*Daphnia* cf. *magna*	Wetland in plateau, Xizang, China	N30°42.442′,E090°53.284′, 4746 m	–	KJ775029	YN2013061703
*Daphnia pulex*	Yuhang, Hangzhou,Zhejiangprovince, China	N30°25.161′,E120°15.666′	–	KJ775030	YN2009102402

N indicates the North latitude, and E indicates East longitude.

### DNA extraction, amplification, and sequencing

Genomic DNA was extracted using a REDExtract-N-Amp Tissue Polymerase Chain Reaction (PCR) Kit (Sigma, St. Louis, MO, USA) according to the manufacturer’s instructions [26]. The mitochondrial COI gene was amplified using the LCO1490 and HCO2198 primers [27]. The nuclear 18S rRNA gene was amplified using the 18 s-F: 5′-AACCTGGTTGATCCTGCCAGT-3′ and 18 s-R: 5′-TGATCCTTCTGCAGGTTCACCTAC-3′ primers from Medlin et al. [28].

The 25-µl PCR reaction consisted of 2 µl of genomic DNA, 8.5 µl of double-distilled H_2_O, 1 µl of each primer (10 mM) and 2× Taq PCR Master Mix (12.5 µl). The thermal conditions used to amplify the COI gene included an initial denaturing step of 5 min at 94°C, 35 cycles of 30 seconds at 94°C, 45 seconds at 51°C, 50 seconds at 72°C, and a final extension of 72°C for 7 min. The thermal conditions used to amplify the 18S gene consisted of two cycles of 30 seconds at 94°C, 45 seconds at 60°C, and 45 seconds at 72°C; five cycles of 30 seconds at 93°C, 45 seconds at 55°C, and 45 seconds at 72°C; followed by 35 cycles of 30 seconds at 93°C, 30 seconds at 50°C, and 3 min at 72°C.

The PCR products were gel-purified and sequenced on an ABI 3730×l sequencer using both the forward and reverse primers. The HZNU collection sequences comprised *S. vetulus*, *S. vetuloides*, *S. beianensis, S. serrulatus*, *S. heilongjiangensis*, *S. himalayensis microdus*, *S. sibiricus*, and *S. himalayensis* from China, *S*. cf. *congener* from Norway, *S. sp.* (“*S. serrulatus”* in Young et al. [25]) from Hangzhou province in China, and *Daphnia* cf. *similoides* from China (Table 1). The nucleotide sequences of the newly analysed specimens were deposited in GenBank database (Table 1).

### Alignment and phylogenetic analyses

We downloaded the available COI sequences from previous studies (*S. congener*, *S. punctatus*, *S.* cf. *punctatus*, *S. vetulus*, *S.* cf. *vetulus*, *S. mixtus*, *S.* cf. *mixtus*, *S. exspinosus*, *S.* cf. *exspinosu*, *S. serrulatus*, *S. heilongjiangensis* and *Simocephalus sp.*) from GenBank (Table 2) and aligned them with our sequences. *Diaphanosoma dubium* (AB549201) and *Daphnia* cf. *similoides* (KF960111) were used as outgroups. The 18S sequences were obtained from the samples in the HZNU laboratory, and *Daphnia* cf. *magna* (KJ775029) and *Daphnia pulex* (KJ775030) were used as outgroups.

**Table 2:** The COI sequences from GenBank that were used in our study.

Species	Genebank accession number	Collection location	Reference
*Simocephalus* cf. *vetulus*	AB549187–AB549193	Taiwan	Young et al. [25]
*Simocephalus vetulus*	KF484582, KF484596, KF484623, and KF484616	Slovakia	Kohout et al. [29]
*Simocephalus* cf. *punctatus* 1	JN233983,JN233988, JN233989, JN233992, and JN233994–JN234003	Canada	Jeffery et al. [24]
*Simocephalus* cf. *punctatus* 1	EU702306–EU702311	Mexico and Guatemala	Elías-Gutiérrez et al. [23]
*Simocephalus* cf. *vetulus*	DQ889172	United Kingdom	Costa et al. [30]
*Simocephalus* cf. *punctatus* 2	JN233976	Canada	Jeffery et al. [24]
*Simocephalus sp.*	KC617418, KC617179, and KC617180	Mexico	Prosser et al. [31]
*Simocephalus* cf. *mixtus* 1	EU702297–EU702301, EU702304, and EU702305	Mexico	Elías-Gutiérrez et al. [23]
*Simocephalus* cf. *mixtus* 2	EU702281	Mexico	Elías-Gutiérrez et al. [23]
*Simocephalus exspinosus*	KF484668 and KF484655	Slovakia	Kohout et al. [29]
*Simocephalus congener*	KF484641 and KF484650	Slovakia	Kohout et al. [29]
*Simocephalus serrulatus*	AB549197 and AB549198	Taiwan	Young et al. [25]
*Simocephalus* cf. *exspinosus* 1	EU702287 and EU702290–EU702296	Mexico	Elías-Gutiérrez et al. [23]
*Simocephalus* cf. *exspinosus* 1	KC617164	Mexico	Prosser et al. [31]
*Simocephalus* cf. *exspinosus* 2	EU702279	Mexico	Elías-Gutiérrez et al. [23]
*Simocephalus heilongjiangensis*	AB549194–AB549196	Taiwan	Young et al. [25]
*Simocephalus serrulatus*	KF484625,and KF484628- KF484630	Slovakia	Kohout et al. [29]
*Simocephalus serrulatus*	JN234006 and JN234007	Canada	Jeffery et al. [24]
*Simocephalus serrulatus*	KC617416 and KC617417	Mexico	Prosser et al. [31]
*Simocephalus serrulatus*	EU702312	Mexico and Guatemala	Elías-Gutiérrez et al. [23]
*Diaphanosoma dubium*	AB549201	Taiwan	Young et al. [25]

The alignment was created using ClustalW [32] and manually edited. The nucleotide composition, conserved sites, variable sites, parsimony-informative sites, transition/transversion ratio, and average genetic distances between each pair of species were determined using MEGA 5.1 [33]. A 658-bp COI fragment and 1976-bp 18S fragment were used for phylogenetic reconstructions. Neighbor-joining (NJ) analyses used the Kimura 2-parameter model with 1,000 bootstraps. Maximum likelihood (ML) analysis which used the GTR+G+I evolutionary model indicated by Modeltest version 3.7 [34], was performed with PhyML V2.4.4 [35] and bootstrap resampled 1,000 times. MrBayes version 3.1.2 [36], [37] was used to generate Bayesian inferences (BI). The program was run for two million generations and sampled every 100 generations, and the first 25% of all of the trees sampled before convergence was discarded as burn-in. The 50% majority rule consensus tree was generated from the remaining trees, and the posterior probability of each node was calculated as the percentage of the trees that recovered the particular node.

## Results

### COI

There were 130 sequences in the alignment of COI sequences. The nucleotide frequencies are 24.0% (A), 38.8% (T/U), 16.5% (C), and 20.7% (G). There are 403 conserved sites, 255 variable sites, and 245 parsimony-informative sites. The overall transition/transversion bias, R, is 1.24. The genetic distances are represented in Table 3.

**Table 3:** The genetic distances (Dxy) between groups of the genus *Simocephalus* based on the COI gene.

Intra-group																					Inter-group	
	1	2	3	4	5	6	7	8	9	10	11	12	13	14	15	16	17	18	19	20	Dxy	SD
1		0.018	0.017	0.019	0.018	0.018	0.018	0.019	0.018	0.018	0.021	0.020	0.019	0.019	0.019	0.019	0.021	0.021	0.021	0.025	0.008	0.003
2	0.163		0.017	0.018	0.011	0.016	0.017	0.019	0.018	0.018	0.021	0.020	0.019	0.020	0.021	0.020	0.020	0.022	0.020	0.027	0.007	0.003
3	0.158	0.146		0.016	0.016	0.016	0.012	0.017	0.018	0.018	0.018	0.018	0.019	0.019	0.020	0.019	0.018	0.021	0.019	0.025	–	–
4	0.160	0.146	0.138		0.017	0.018	0.016	0.018	0.020	0.019	0.021	0.021	0.020	0.021	0.020	0.021	0.019	0.020	0.019	0.023	0.000	0.000
5	0.159	0.070	0.132	0.137		0.016	0.015	0.017	0.017	0.017	0.020	0.019	0.018	0.019	0.020	0.020	0.020	0.021	0.018	0.024	0.000	0.000
6	0.160	0.139	0.127	0.161	0.132		0.016	0.018	0.018	0.019	0.019	0.019	0.020	0.020	0.020	0.020	0.019	0.020	0.020	0.024	0.000	0.000
7	0.161	0.143	0.075	0.136	0.134	0.135		0.018	0.018	0.017	0.018	0.018	0.017	0.020	0.020	0.018	0.019	0.022	0.019	0.025	0.025	0.005
8	0.182	0.169	0.152	0.165	0.143	0.169	0.161		0.017	0.017	0.018	0.017	0.017	0.019	0.018	0.017	0.018	0.021	0.020	0.023	0.020	0.004
9	0.173	0.173	0.165	0.190	0.158	0.171	0.166	0.159		0.012	0.014	0.014	0.012	0.018	0.015	0.014	0.019	0.022	0.020	0.027	0.000	0.000
10	0.174	0.177	0.171	0.183	0.164	0.179	0.159	0.155	0.070		0.014	0.014	0.005	0.018	0.016	0.016	0.019	0.022	0.020	0.025	0.000	0.000
11	0.209	0.209	0.171	0.207	0.194	0.190	0.170	0.171	0.106	0.018		0.006	0.015	0.020	0.017	0.016	0.020	0.022	0.022	0.028	0.000	0.000
12	0.194	0.194	0.176	0.198	0.191	0.187	0.170	0.161	0.108	0.106	0.019		0.014	0.019	0.017	0.015	0.019	0.021	0.020	0.027	0.000	0.000
13	0.180	0.180	0.176	0.189	0.167	0.184	0.160	0.154	0.076	0.016	0.116	0.110		0.019	0.016	0.016	0.019	0.022	0.020	0.025	0.031	0.007
14	0.178	0.196	0.192	0.194	0.177	0.196	0.193	0.166	0.159	0.169	0.191	0.179	0.168		0.020	0.020	0.021	0.021	0.020	0.026	0.023	0.004
15	0.193	0.208	0.195	0.200	0.187	0.194	0.200	0.162	0.126	0.132	0.145	0.143	0.133	0.192		0.016	0.019	0.020	0.022	0.027	0.000	0.000
16	0.190	0.193	0.186	0.206	0.199	0.194	0.178	0.158	0.107	0.128	0.131	0.114	0.126	0.197	0.130		0.018	0.021	0.020	0.027	0.007	0.003
17	0.196	0.204	0.182	0.184	0.191	0.174	0.183	0.164	0.168	0.184	0.182	0.180	0.183	0.202	0.171	0.172		0.022	0.020	0.025	0.007	0.003
18	0.201	0.210	0.211	0.181	0.197	0.175	0.214	0.198	0.224	0.209	0.216	0.206	0.212	0.203	0.187	0.217	0.217		0.022	0.025	0.032	0.007
19	0.215	0.211	0.188	0.186	0.183	0.193	0.189	0.200	0.190	0.183	0.208	0.199	0.188	0.198	0.234	0.188	0.189	0.208		0.023	–	–
20	0.286	0.310	0.290	0.255	0.285	0.272	0.278	0.256	0.306	0.286	0.316	0.302	0.283	0.278	0.303	0.317	0.279	0.295	0.257		–	–

1–20 indicate groups of *Simocephalus*: 1, *Simocephalus* cf. *vetulus* from China and Taiwan; 2, *S. vetulus* from Europe; 3, *Simocephalus* cf. *vetulus* from Europe; 4, *S. vetuloides* from China; 5, *S. beianensis* from China; 6, *S. sp.* from North America; 7, *S.* cf. *punctatus* from North America; 8, *S.* cf. *mixtus* from North America; 9, *S. himalayensis* from China; 10, *S.* cf. *congener* from Europe; 11, *S. congener* from Europe; 12, *S. himalayensis microdus* from China; 13, *S. exspinosus* from Europe; 14, *S.* cf. *exspinosus* from Europe; 15, *S. sibiricus* from China; 16, *S.sp* = *S. serrulatus* form Taiwan in Young et al. 2012; 17, *S. heilongjiangensis* from China and Taiwan; 18, *S. serrulatus* from North America, China and Europe; 19, *Daphnia* cf. *similoides* from China (outgroup); 20, *Diaphanosoma dubium* from Taiwan (outgroup).

Intra-group and inter-group genetic distances of the 20 groups involved 132 nucleotide sequences. The standard deviations (SD) are shown in the upper diagonal of the matrix and the right column. Analyses were conducted using the Kimura 2-parameter model.

**Figure 1:**
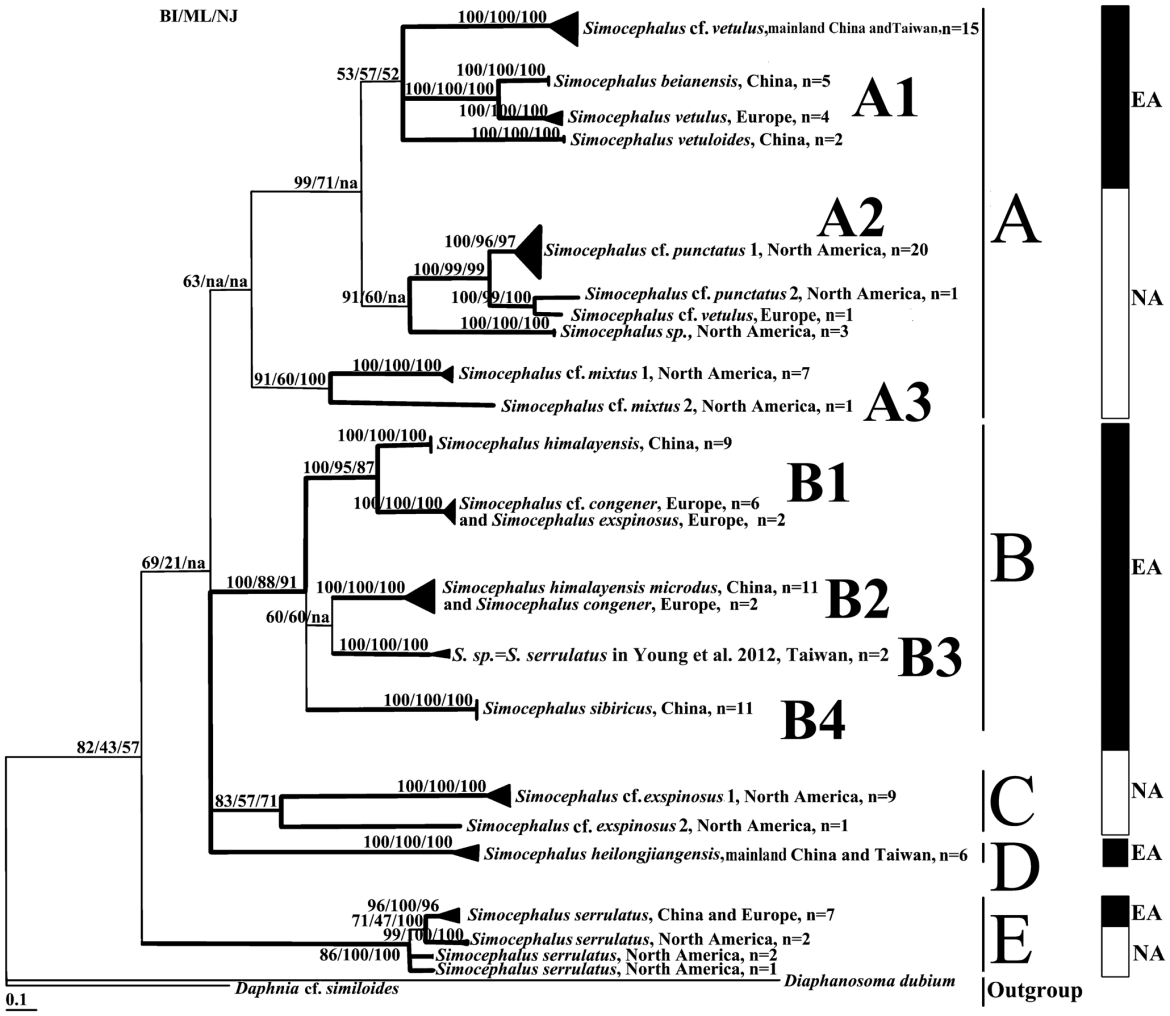
The phylogeny of *Simocephalus* inferred from mitochondria cytochrome *c* oxidase subunit I (COI) sequences as a consensus tree formed from trees constructed using Bayesian inference (BI), maximum likelihood (ML), and neighbor-joining (NJ) methods. Numbers at nodes are as follows: BI posterior probability value multiplied by 100 for legibility, followed by bootstrap values between 0 and 1 from ML and NJ analyses. The scale bar corresponds to 0.1 substitutions per nucleotide position. In the left column, EA indicates Eurasian, and NA indicates North American. A, B, C, D, and E indicate the five subgenera. A: *Simocephalus* s. str.;B and C: *Simocephalus* (*Echinocaudus*); D: *Simocephalus* (*Aquipiculus*); and E: *Simocephalus* (*Coronocephalus*).

The NJ, ML, and BI phylogenetic analyses led to highly congruent tree topologies (Fig. 1). In all of the trees, the terminal branches represent 100% support for presumed biological species of *Simocephalus*. Sometimes such biological species are undistinguishable if morphological identification is used (see for example *Simocephalus congener* and *S.* cf. *congener* in Europe, Fig. 1A) Their number is not fully clear, as indicated by clades A2 and E, for example. Although the statistical support for the deep branches is low, the grouping of the deeper clades generally agrees with the intra-generic classification of Orlova-Bienkowskaja [9], namely for *Simocephalus* (*Echinocaudus*), *S.* (*Coronocephalus*), *Simocephalus* s. str., and *S.* (*Aquipiculus*).

Clade A represents *Simocephalus* s. str., and it contains two subclades, A1 (*S.* cf. *vetulus* from mainland China and Taiwan, *S. beianensis* and *S. vetuloides* from China, and *S. vetulus* from Europe) and A2 from North America (*S.* cf. *punctatus* 1 and 2, *S.* cf. *vetulus* and 2 *Simocephalus sp.*, KC617180 and KC617418). Subclades A1+ A2 are grouped with subclade A3 (*S.* cf. *mixtus* 1 and 2 from North America), but this has very low support. *Simocephalus* s. str. (clade A) is resolved as the sister group to *Simocephalus* (*Echinocaudus*) (clades B+C) and *S.* (*Aquipiculus*) (clade D), but support was also very low.

Clade B is the Eurasian portion of *Simocephalus* (*Echinocaudus*). It contains sub*-*clade B1 (*S.* cf. *congener* and *S. exspinosus* from Europe, most likely a single taxon, and *S. himalayensis*) and has high posterior probability and high bootstrap support (BI/ML/NJ, 100/95/87). The other clade, B2, contains *S. congener* from Europe and *S. himalayensis microdus* (as a subspecies whose separate status is questionable). Clade B3 consists of “*S. serrulatus*” from Taiwan. The fourth clade (B4) contains only *S. sibiricus.* It is important that all of the Eurasian taxa of *S*. (*Echinocaudus*) form a monophyletic group that is well-supported (BI/ML/NJ, 100/88/91, respectively) by different statistical analyses.

Clade C, the American portion of *Simocephalus* (*Echinocaudus*), contains two taxa, *S.* cf. *exspinosus* 1 and 2 from North America, with moderate support.

In this analysis, clade D, *S.* (*Aquipiculus*), contains only a single taxon, *S. heilongjiangensis.*

Clade E, *S.* (*Coronocephalus*), contains various clades of *S*. *serrulatus* from North America and Eurasia, and the number of taxa in this complex is unclear. *S.* (*Coronocephalus*) is resolved as basal to other species of *Simocephalus* that are distant one from another, but support for this position is relatively weak.

The genetic distances between groups which were formed by the sequences were calculated (Table 3). The intra-group genetic distance of *Simocephalus* varies from 0.070 to 0.224, and the inter-group genetic distance of *Simocephalus* is not exceeding 0.008. The greatest genetic distance is between *S. himalayensis* and *S. serrulatus*, while the smallest is between *Simocephalus* cf. *congener* and *S. himalayensis* (7.0%).

### 18S

Ten sequences of 18S were obtained, the nucleotide frequencies are 21.5% (A), 38.8% (T/U), 24.5% (C), and 30.3% (G). There are 1604 conserved sites and 345 variable sites, of which 212 are parsimony-informative. The overall transition/transversion bias, R, is 0.75.

**Figure 2:**
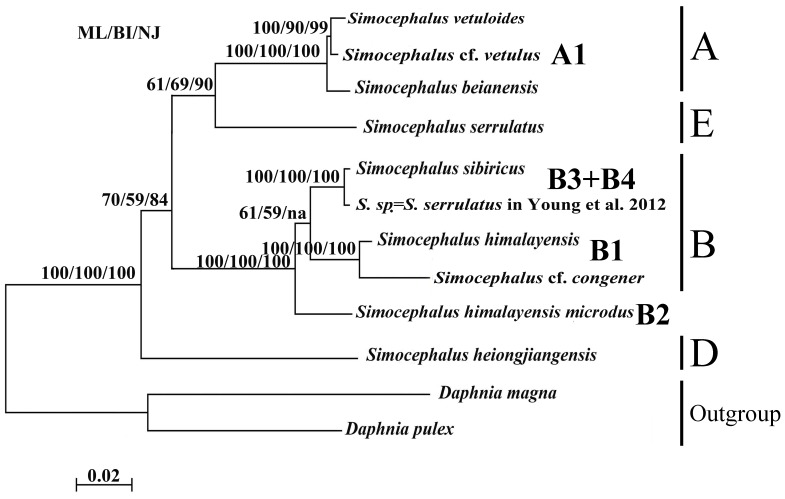
Phylogeny of *Simocephalus* inferred from 18S sequences as a consensus tree formed from trees constructed using maximum likelihood (ML), Bayesian inference (BI), and neighbor-joining (NJ) methods. ML bootstrap values between 0 and 1, followed by BI posterior probability value multiplied by 100 for legibility and bootstrap values between 0 and 1 from NJ analyses. The scale bar corresponds to 0.02 substitutions per nucleotide position. A indicates *Simocephalus* (*Echinocaudus*), B *Simocephalus* s. str., D *Simocephalus* (*Aquipiculus*), and E *Simocephalus* (*Coronocephalus*).

The tree (Fig. 2) contains four well-supported clades (A, B, E, D) that correspond to the subgenera identified by Orlova-Bienkowskaja [9]. As in the case of COI, “*S. serrulatus”* from Young et al. [25] appears within the *S*. (*Echinocaudus*) subgenus, which confirms the misclassification of this specimen. *Simocephalus* s.str. (clade A, abbreviations as in the COI tree) is represented only by the subclade A1. It is a sister group of *S*. (*Coronocephalus*) (clade E), and the clade containing these two subgenera is a sister group to *S*. (*Echinocaudus*) (clade B) which contains clades B1, B2 and B3+B4, corresponding to the clades from the COI tree. *S.* (*Aquipiculus*) (clade D) is the basal-most taxon of the genus *Simocephalus* in this analysis with strong statistical support for this position. No representative of clade D from the COI tree is present in this tree. See the genetic distances in Table 4.

**Table 4:** The genetic distances (Dxy) between each pair of species of genus *Simocehalus* involved 12 nucleotide sequences include outgroups are shown based on the 18S gene.

	1	2	3	4	5	6	7	8	9	10	11	12
1		0.003	0.002	0.005	0.005	0.005	0.005	0.004	0.005	0.004	0.006	0.006
2	0.007		0.003	0.005	0.005	0.005	0.005	0.005	0.005	0.005	0.006	0.007
3	0.008	0.012		0.004	0.005	0.005	0.005	0.005	0.005	0.004	0.006	0.006
4s	0.046	0.050	0.043		0.003	0.003	0.003	0.003	0.005	0.005	0.007	0.007
5	0.047	0.051	0.045	0.006		0.003	0.004	0.004	0.005	0.006	0.007	0.006
6	0.047	0.051	0.046	0.019	0.018		0.003	0.003	0.005	0.005	0.007	0.006
7	0.049	0.052	0.047	0.020	0.022	0.023		0.002	0.005	0.005	0.007	0.007
8	0.043	0.049	0.043	0.015	0.017	0.018	0.007		0.005	0.005	0.007	0.007
9	0.044	0.050	0.044	0.061	0.060	0.061	0.063	0.057		0.005	0.006	0.006
10	0.032	0.038	0.036	0.054	0.056	0.057	0.056	0.050	0.055		0.006	0.006
11	0.080	0.085	0.082	0.087	0.087	0.089	0.093	0.088	0.089	0.084		0.005
12	0.076	0.082	0.076	0.070	0.079	0.082	0.084	0.078	0.086	0.082	0.052	

1-12 indicate: 1, *Simocephalus* cf. *vetulus*; 2, *S. vetuloides*; 3, *S. beianensis*; 4, *S. himalayensis*; 5, *S.* cf. *congener*; 6, *S. himalayensis microdus*;

7, *S. sibiricus*; 8, *S. sp.* = *S. serrulatus* in Young et al.2012; 9, *S. heilongjiangensis*; 10, *S. serrulatus*; 11, *Daphnia magna*; 12, *D. pulex*.

The standard deviations (SD) are shown in the upper diagonal. Analyses were conducted using the Kimura 2-parameter model.

The position of clade E differs between the 18S and COI trees. In both cases statistical support of its grouping with other branches is moderate or definitively insufficient for a final verdict. Therefore we need additional studies (using other genes?) for understanding the exact position of *S*. (*Coronocephalus*) in the genus.

## Discussion

Our study confirms the opinion [1] that a real diversity of the cladocerans is several times higher than is accepted now, owing to the existence of many cryptic species complexes instead of “traditional” taxa. Our study also supported the concept of “continental endemism” [6], [38]. In the case of *Simocephalus*, only the populations of *S. serrulatus* from Europe and North America seem to be closely related; there are no other species shared between the two continents. We propose that the differentiation of some clades, such as the Eurasian and North American sections of *S*. (*Echinocaudus*), most likely each took place within the continent to which they are now largely confined.

In the case of *Simocephalus*, the COI barcoding approach was very effective for the discrimination of cryptic species. This might be explained by the age of the genus which is known since the Mesozoic [39], [40]. Each subgenus of *Simocephalus* has recent taxa on different continents (except Antarctica), which could be regarded as confirmation of an ancient, possibly Mesozoic, differentiation between subgenera that occurred before the continental break up, similarly to the subgenera of *Daphnia* [40]. We believe that the continental endemism of *Simocephalus* taxa is also mainly explained by their old age. At the same time, we also found some cases of later, inter-continental, differentiation, see above.

According to the rule-of-thumb of the barcoding approach [41], two clades are considered as distinct species if the divergence between them in COI sequences is greater than 3% while lower (0.7–2.2%) values suggest recent divergence of a clade. Of course, these values seem to vary in different groups of the Daphniidae; the mutation rate is much faster in halophilic cladocerans, for example [41], [42]. However, all of the terminal branches revealed by the 3% criterion are potentially separate species, which are thereby quite numerous in China.

In many cases, appropriately naming such taxa is impossible. Due to greater, recent activity by molecular phylogeneticists in North America [23], [24], [31], the continent is simply better studied. In contrast, type localities of the majority of the “non-Chinese” species are located in Europe (*S. vetulus, S. congener, S. exspinosus,* and *S. serrulatus*) or Eastern Siberia (*S. mixtus, S. vetuloides,* and *S. sibiricus*). These regions have not been adequately studied genetically except in the preliminary work of Kohout et al. [29] in Central Europe.

Orlova-Bienkowskaja [9] proposed to differentiate the five subgenera within the genus *Simocephalus* based on the shape of the frontal part of the head, rostrum shape, ocellus shape, length of the postero-dorsal valve prominence, expression of the pre-anal angle, anal teeth on postabdomen and presence of basal or distal pecten of spines on the postabdominal claw. In our study, the COI and 18S trees support this classification. As usual, the statistical support for the deeper branches of the COI tree is insufficient to draw any conclusions [23].

The characters used by Orlova-Bienkowskaja [9] for species discrimination are less successful as noted earlier by Hann [43]. Some characters seem to be too variable and originate many times in different clades, such as:

1. Ocellus shape. This character was found to be very variable even in a single population of *S. vetulus* [44]. A minute ocellus appears several times in the evolution of the genus, see Fig. 1, clade A2.
2. Shape of the postero-dorsal valve prominence. Earlier, Young et al. [25] showed that *S. vetulus, S. mixtus* and *S. vetuloides*-like morphotypes from Taiwan belong to a single species, and the size of the postero-dorsal prominence is too variable to be used in the taxonomy of, at least, this clade. In our tree, there are two clades conforming to the diagnosis of *S*. cf. *vetulus* in clade A1. Therefore, the shape of the postero-dorsal prominence does not work well for species determination.
3. Size and number of spines in the basal pecten on the postabdominal claw. According to the species determination scheme of Orlova-Bienkowskaja [9] the main differences between *S. exspinosus* and *S. congener* concern the anal teeth and the basal pecten of the spines on the postabdominal claw. *Simocephalus exspinosus* has 12 to 22 teeth while *Simocephalus congener* bears 9 to 18 teeth, according to Orlova-Bienkowskaja [9] and 7 to 9 in our material. The former has 8 to 12 moderately-sized postabdominal spines while the latter has 20 to 25 fine spines or 18 according to Orlova-Bienkowskaja [9] and 17 to 20 in our material from Norway. Earlier, Hann [43], based both on morphology and the electrophoretic analysis of allozymes, proposed that there are “*S. exspinosus”* and “*S. congener”* hybrids in Canada. In addition, the spectra of variability seem to overlap. Therefore, the significance of the size and number of the spines in the basal pecten must be regarded as unknown to date. In our tree, *S. exspinosus* and *S.* cf. *congener* from Europe look to belong to a single taxon (clade B1) in contrast to other, morphologically similar, forms, such as *S. himalayensis microdus* from China, *S. congener* from Europe (clade B2), and others. There are even two *congener*-like taxa in Europe, clades B1 and B2.

**Figure 3:**
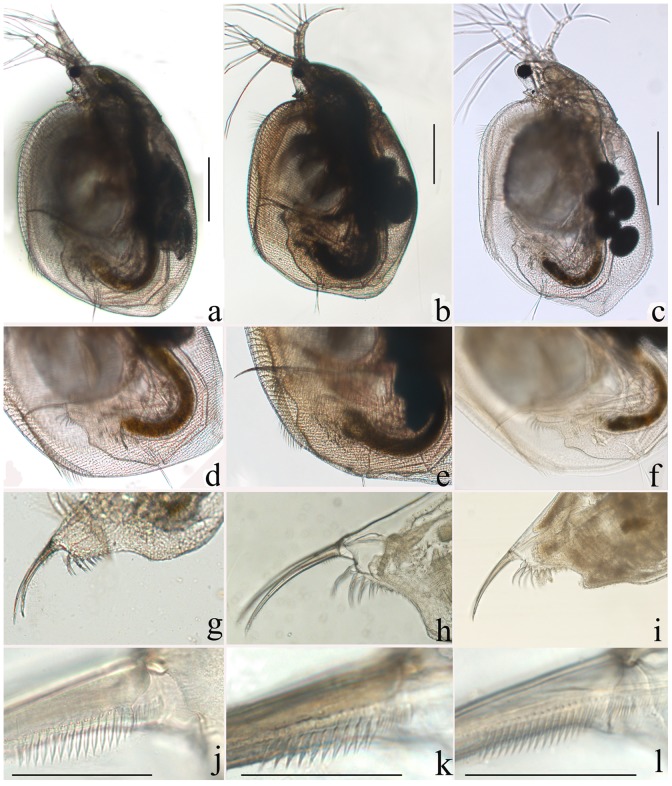
Photographs of living parthenogenetic females of the three morphologically-similar species from China: *S. himalayensis microdus* (a, d, g, j), *S. himalayensis* (b, e, h, k), and *S. sibiricus* (c, f, i, l). General view (a–c). General view of the postabdomen (d–f). Distal portion of the postabdomen (g–i). Pecten of spines on the postabdominal claw (j–k). Scale bars: 0.5 mm (a–i); 0.01 mm (j–l).

Orlova-Bienkowskaja [9] proposed that *S. sibiricus* and *S. himalayensis* are junior synonyms of *S. exspinosus*. In contrast, Chen et al. [14] and Shi et al. [18] found some differences among *S. himalayensis*, *S. himalayensis microdus*, and *S. exspinosus*. Table 5 summarizes the differences between the taxa of the *Simocephalus* (*Echinocaudus*) subgenus in China based on information from Chinese sources (see also Fig. 3). Unfortunately, most of these “differences” are very dubious and appear to have been proposed despite insufficient information on the variability in such characters throughout the whole Eurasian range. Characters such as presence-absence of small teeth on the anal embayment and the expression of the preanal angle of the postabdomen seem to be more promising (Fig. 3), but variability in the former and the latter must be studied. We believe that male characters could be more important for taxonomy, but they have not yet been adequately described.

**Table 5:** Main differences among five species of *Simocephalus* (*Echinocaudus*).

Species	*S.* cf. *congener* in oursample from Norway	*S. exspinosus*	*S. sibiricus*	*S. himalayensis*	*S. himalayensis microdus*
Maximum body length	1.62±0.13	2.15±0.27	1.73±0.17	3.10±0.10	2.64±0.23
Postero-dorsal valve, shape and prominence	Rounded, absent	Small or absent	Small, obvious	Small	Small
Ocellus	Short rhomboid	Point-like	Short rhomboid	Point-like	Round or rhomboid
Anal teeth	7–10	12–22	10–16	12–14	11–19
Basal pecten of spines	17–20	8–12	16–22	12–15	8–21
Posterior valve margin	Dorsal posterior valvemargin with small denticles	The dorso and ventral posteriorvalve margin with small denticles	Dorsal posterior valve marginwith thick and strong denticles	Smooth, without denticle	Smooth, without denticle
Posterior anal angle	Pointed and protruding	Not obviously protruding	Pointed and protruding	Not protruding	Not protruding
References	This work	Shi et al. [18]	Shi et al. [18]	Shi et al. [18]	Shi et al. [18]

## Conclusion

Our study unambiguously confirmed the existence of both local and widely distributed lineages from the subgenera of *S.* (*Echinocaudus*) and *Simocephalus* s.str. in China. To date, their determination based on morphological characters is difficult. But it is a consequence of their inadequate study instead of morphology “lacking resolution” [41]. Morphology of different cladoceran taxa needs to be reexamined by taking a wider range of characters into consideration (e.g., of female thoracic limbs and of adult males). However, keeping in mind that many species were previously described using European populations as the type specimen, a new revision of the European taxa that combines molecular and morphological methods is also urgently needed.
